# Strategies for Group-Level Mentoring of Undergraduates: Creating a Laboratory Environment That Supports Publications and Funding

**DOI:** 10.3389/fpsyg.2019.00323

**Published:** 2019-02-21

**Authors:** Amy A. Overman

**Affiliations:** Department of Psychology & Neuroscience Program, Elon University, Elon, NC, United States

**Keywords:** mentoring activities, shared vision, cohort building, interlocked projects, undergraduate research, grant writing, peer mentoring

While one-on-one mentoring relationships (Shellito et al., 2001) and institution-level support for mentoring (Rowlett et al., 2012) are essential aspects of undergraduate research, little emphasis has been placed on developing mentoring strategies at the level of the research group or laboratory. Many of us, especially in the sciences, mentor several undergraduate students in research during a semester and over multiple years. Some, or all, of the students may be working on shared or related projects. Successful mentoring of students requires constructive relationships at both the individual and group levels (Shanahan et al., 2015). However, the literature on group-level mentoring primarily focuses on student outcomes, rather than on how group-level mentoring benefits *faculty* by creating an infrastructure for producing publishable research and securing external funding.

This article describes several strategies for group-level mentoring of undergraduates to foster research productivity and simultaneously provide valuable high-impact educational experiences for students (Kuh, 2008). These strategies can be considered forms of cohort building, which has been shown to support positive outcomes for students in STEM fields, including an increased sense of belongingness (Gross et al., 2015). Belongingness, in turn, is an important aspect of motivation (Baumeister and Leary, 1995) and of student academic performance and health (Ames, 1992; Walton and Cohen, 2011), and is a prominent topic in higher education with regard to student retention, graduation rates, and transformative learning (Felten et al., 2016). In my experience, applying cohort-building practices at the laboratory group level also increases faculty productivity. In the following sections, I describe three types of strategies for group-level mentoring and provide a description of how each strategy supports the tangible research outcomes of publications and external funding.

## Shared Vision

Shared vision is a common management concept (e.g., Kouzes and Posner, 2009), and many readers have some experience with generating or maintaining vision statements at their institutions. A shared vision results in more investment and work satisfaction (Slack et al., 2010), yet we often do not take the time to construct a shared vision with the undergraduate researchers we mentor. As novices, undergraduates may know what hypotheses are being tested and what other researchers have published on a topic but fail to understand the value of the study to the field and how it contributes to long-term goals in our own faculty research agenda.

There are two important pieces of developing shared vision: developing students' personal vision of themselves as scientific researchers, and aligning that personal vision with the larger-scale group vision for the lab's research, including how that research contributes to the field and to society. Here, I describe activities that can shape both aspects of shared vision.

### Promoting a Sense of Scientific Identity

Effective personal vision is thought to be based on an “ideal self,” which includes the person's core identity (Boyatzis et al., 2015). Sustained undergraduate research participation fosters scientific identity (Linn et al., 2015), and mentors can enhance this development through simple habits such as frequently and explicitly talking with students about their identity as scientists and the nature of science, and assigning activities that require students to reflect on these topics. For example, I assign students a reading about core traits of successful scientists from *Science* magazine's website (Jensen, 2018), and students write about how they have already demonstrated, and will demonstrate, those traits in their work as a researcher in my lab. Through such assignments and conversations, I have seen students internalize a scientific identity that helps them to fully embrace the research vision of the lab.

Likewise, the group's larger vision can be strengthened through frequent and explicit reinforcement of how specific research projects in the laboratory serve a greater purpose. Research suggests that the alignment of personal and group visions in this way supports greater commitment and engagement (Berg, 2015). One way I iteratively encourage this alignment each semester is to ask each student to complete an undergraduate research mentoring agreement. This agreement includes specific details about the lab's research vision and how the student is expected to support that vision during the semester.

### Encouraging Shared Vision Through Grant Writing

An especially powerful way to solidify shared vision in students' minds is by incorporating mentored students into the grant-writing process. Grant proposals are a rallying point for students, making explicit how their own research findings benefit society, and giving them a tangible way to “root for” the laboratory in the competitive funding process. Students in my lab participate in writing their own internal and external grant applications and have been part of the process when I applied for external funding.

Students are usually unfamiliar with grants, so I encourage a grant-seeking mindset by explicitly discussing the role of funding in the advancement of research, sharing undergraduate funding opportunities with them, and providing feedback on their applications. Throughout this process, students must articulate why their research matters and how it is valuable in advancing the field. Students in my lab have applied for, and been awarded, competitive external summer research fellowships as well as external funding for their research projects via organizations such as Psi Chi.

When I was writing the application for my current National Institutes of Health R15 award, I regularly discussed the application process with my mentored students. They were unfamiliar with the NIH, so I assigned portions of the NIH website and program announcement for them to read, and asked them to write about what they learned. We discussed the criteria by which the grant would be evaluated, and the students gave feedback on what I'd written. Students also contributed to the pilot studies that were included in the grant, which increased their sense of accomplishment and excitement, in addition to helping move those studies to publication.

### Benefits to Research Productivity

Effectively fostering shared vision in the laboratory has numerous concrete benefits to research productivity. One of the greatest benefits is that shared vision helps motivate students to work on projects that support and extend existing lines of research. This is crucial in establishing a record of publication in a concentrated area, which is necessary for securing external funding. Additionally, students who embrace a scientific identity are more likely to participate long-term, invest, and take ownership of their projects (Hanauer et al., 2012), increasing the likelihood of producing high-quality publications. Furthermore, getting students involved and excited about the process of competing for funding can provide a motivational boost to the faculty member in getting proposals initiated and submitted. Finally, receiving grant funding has been shown to increase faculty co-authorship of publications with undergraduates (Morales et al., 2017).

## Interlocking Projects

A second major strategy I employ is to guide students' interests in a way that creates intersections among projects in the lab while preserving students' agency in developing their own ideas. When students express interest in conducting research, I ask them to describe how their strengths align with the work of the lab and to identify what particularly appeals to them and why. Then, I outline possibilities for new projects that connect research areas in the lab. If we begin a mentoring relationship, the student, with guidance, develops a project that lives in the space between existing projects, and interlocks with them like a missing puzzle piece. For example, one current student's project focuses on how word generation affects young adults' memory for contextual information. This project connects to another project focused on older adults' memory for context and to another project investigating item-specific aspects of generation effects in memory. The interconnectedness amongst projects allows me to cross-train students and allows students to share knowledge with each other because there is always at least one similarity between two projects that provides common ground.

### Structuring Projects for Peer Mentoring

An additional element of the interlocking-project strategy is intentionally arranging peer-mentoring matches between newer and experienced lab members. The benefits of peer mentoring in research are well-documented (Lopatto, 2010; Packard et al., 2014). Because of the interlocked nature of lab projects, I can assign peer mentors primarily based on skill set, rather than according to the project topic. The 2-fold goal is to scaffold skill learning for the newer member and to allow the experienced lab member to serve as a resource and guide. Peer mentors meet with me to discuss elements of good mentoring, the specific goals for the peer mentoring relationship (e.g., understanding research processes and tasks, imparting lab culture, personal development), and how best to reach those goals. For example, if an experienced student is particularly good at data analysis, I partner her with a student who has less analysis experience. The newer student shadows several times before moving on to conduct his own data analysis, while being observed by the experienced student who then gives feedback to him on how he performed. This happens in parallel with my own direct guidance and feedback to the student who is learning the new skill and to the peer mentor regarding their mentoring. Experienced students consistently report that it helps them realize how much they have learned as a researcher and that they enjoy sharing knowledge and tips for success. Newer students consistently report how helpful it is to learn from someone who was relatively recently at their current stage, and how partnering with the experienced student helped them feel more confident in their ability to learn and more connected to the lab as a whole.

### Benefits to Research Productivity

Interlocking projects and peer mentoring extend the benefits of the shared vision strategy. In addition to broadly investing in a common set of goals and ideas, students also focus their specific work on closely-related skills, concepts, and scientific questions, which enables the faculty mentor to lead them collectively to produce more sustained and higher-impact research than would be possible through one-off projects with individual students. I have guided students' parallel projects with the aim of incorporating several of them into a multi-experiment paper, and sequential projects to create a series of publications on a single topic. Peer mentoring that is facilitated by an interlocking project structure also enables new students to move projects toward publication more efficiently than if they were solely mentored by the faculty member.

## Lab Community

Shared vision and interlocking projects create a strong foundation for lab community and productivity, which is cemented through intentional investment in the social facets of working together. The role of social interaction and support in building a sense of community is well-documented (Hoffman et al., 2002) but prior literature on community-building in research mentoring has focused on institution-level practices (Bender et al., 2008).

One way I intentionally work to strengthen relationships among students is to assign students to share coffee or a meal with someone in the lab with whom they do not collaborate. They are instructed to share details of their projects, similarities and differences between their projects, what they hope to learn during their time in the lab, what is most valuable that they've already learned, and what advice they would give new researchers. Lastly, they are required to discuss two things unrelated to research. Students consistently report surprise and delight at the common ground they discover with their peers.

### Benefits to Research Productivity

Beyond the inherent benefits to students' development and well-being, strengthening community through relationship-oriented interactions enhances productivity in the research laboratory. The sense of belongingness bolsters motivation when students hit inevitable low points in the research process. Additionally, lab friends are a resource that students can access when their own problem-solving hits a wall, which reduces the demand on the faculty member to address smaller issues like coding or analysis snags. Both of these effects help students to work independently and keep projects moving toward completion and dissemination with less prompting from the faculty mentor.

## Conclusion

Group-level strategies can be implemented to successfully engage undergraduates in research that is high-quality and likely to be published and funded. The aforementioned strategies and activities are aimed at the collective development and organization of the research group or whole laboratory. They can also be adapted to research groups that include graduate students, or to teams of student researchers being led by a graduate student or post-doctoral researcher within a larger research lab. When thoughtfully applied, these strategies can help mentors to develop high-impact, productive research programs with undergraduate students.

## Author Contributions

The author confirms being the sole contributor of this work and has approved it for publication.

### Conflict of Interest Statement

The author declares that the research was conducted in the absence of any commercial or financial relationships that could be construed as a potential conflict of interest.
